# Women's family planning status in Ethiopia: A geo-additive modeling approach

**DOI:** 10.1016/j.gloepi.2026.100275

**Published:** 2026-06-19

**Authors:** Mandefro Abere Tegegne, Essey Kebede Muluneh, Aweke Abebaw Mitku, Girma Taye Aweke

**Affiliations:** a Department of Statistics, College of Sciences, Bahir Dar University, P.O. Box 79, Bahir Dar, Ethiopia; b Department of Statistics, College of Natural and Computational Sciences, University of Gondar, P.O. Box 196, Gondar, Ethiopia; c Department of Epidemiology and Biostatistics, College of Medicne and Health Sciences, Bahir Dar University, P.O. Box 79, Bahir Dar, Ethiopia; d Department of Epidemiology and Biostatistics, College of Health Sciences, Addis Ababa University, Addis Ababa, Ethiopia; e Global Change Institute (GCI), Faculty of Science, University of the Witwatersrand, South Africa

**Keywords:** Geo-additive modeling, Family planning, Contraceptive use, Unmet needs, Spatial effect, Women, Ethiopia

## Abstract

**Background:**

A complex interplay of socioeconomic, cultural, and geographic factors influenced women's family planning status in Ethiopia, resulting in significant zonal disparities. Traditional statistical models often fail to capture adequately the nonlinear and spatial components underlying these choices.

**Objective:**

The study aimed to predict and assess the spatial, linear, and nonlinear effects of factors associated with women's family planning status in Ethiopia zones.

**Methods:**

The study implemented a multi-stage cluster design and a geo-additive model. We drew secondary data from 7484 women of reproductive age, collected by the Performance Monitoring and Accountability (PMA2020) project.

**Results:**

The geo-additive approach performed better than linear and additive models. Contraceptive use and unmet needs varied across zones, with spatial structure explaining 93% and 68% of the variation, respectively, confirming substantial spatial autocorrelation. Contraceptive use was more likely among those with higher education, higher socioeconomic status, fewer children, more health center visits, and a lower desire for more children. Unmet needs were associated with desire for children, sex avoidance, and the use of private healthcare facilities. Continuous variables have substantial and nonlinear effect.

**Conclusions:**

The study's findings suggest that spatial and nonlinear variables affect women's family planning status. The spatial patterns closely aligned with the distribution of key predictors, such as education, wealth, parity, health center access, and fertility preferences. Consequently, reproductive health policies and programs in Ethiopia should be designed with attention to individual-level determinants and spatial disparities to ensure more equitable and effective outcomes on family planning.

## Introduction

In Ethiopia, socioeconomic, cultural, and educational variables impact women's family planning (FP) choices. Despite the recognition of women's autonomy in reproductive health decisions, contraceptive use remains low, with only about 41.4% of women using any contraception, indicating a significant unmet demand for FP services [1], [2]. Several obstacles women confront, including limited access to information, socio-cultural norms, and financial restraints, aggravated this scenario.

Besides these individual and social factors, geographic disparities significantly affect FP utilization. Urban areas tend to have better access to FP services than rural areas, where traditional beliefs may be more entrenched and access to healthcare facilities is limited. Studies reveal regional disparities in Ethiopian women's family planning decisions, with location explaining almost 25% of the variation in modern contraceptive use and unmet needs [3], [4].

Understanding the rationale behind women's FP choices thus requires a lens that captures these regional disparities For example, women may prioritize limiting family size in some zones because of economic pressure or better awareness. Conversely, in other cultures, people value larger families and discourage the use of contraceptives. Access to FP information and services can also vary drastically by zone due to differences in healthcare infrastructure, outreach efforts, and policy implementation.

Ignoring the impact of location when modeling women's family planning decisions will result in inaccurate conclusions, as nearby areas often share similar socioeconomic or cultural backgrounds. [5], [6]. In Ethiopia, traditional linear models violate the assumption of spatial independence because neighboring areas often share identical cultures and services. Location explained a substantial portion of the variation in contraceptive use, according to prior studies, emphasizing the importance of models incorporating these spatial effects.

Structural Additive Regression Models (STAR) present a compelling solution to these limitations. It provides a flexible framework for modeling possible nonlinear effects of covariates and geographic impact. As an extension of Generalized Additive Models (GAMs), STARs employ smooth functions to flexibly model both fixed and nonlinear effects of predictors while accommodating interactions between continuous and categorical variables [7], [8], [9]. This adaptability is advantageous in contexts where continuous determinants exhibit nonlinear relationships with women's FP choice [10], [11], [12]. Although structural additive regression models have strong methodological potential, they remain underutilized in FP research, particularly for modeling unordered multinomial outcomes with spatially structured regional effects. Additionally, empirical studies and data at the zonal level in Ethiopia are insufficient. Unlike conventional logistic regression, our methodology simultaneously accounts for nonlinear effects and spatial autocorrelation, uncovering zonal differences that would otherwise be hidden.

Therefore, this study applies STAR models with spatially structured random effects and nonlinear smoothing terms to reveal how multiple factors influence family planning decisions. Policymakers can use this modeling approach to identify areas with the most significant spatial disparities and create customised solutions to address individual and neighbourhood challenges.

## Methods

### Data and study settings

**Fig. 1:**
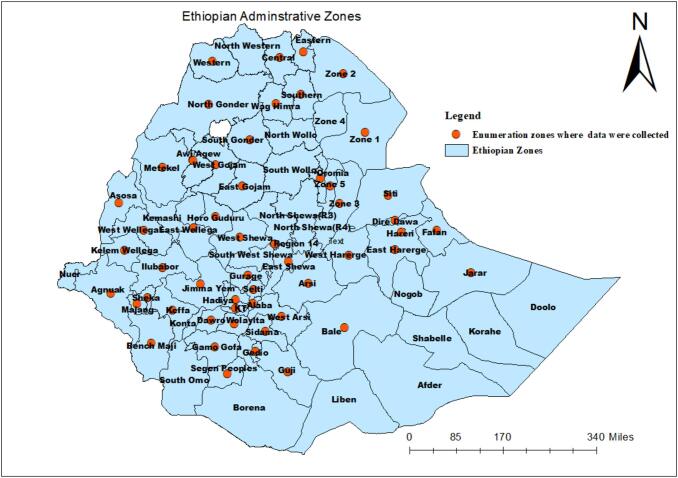
Map of Ethiopian administrative zones included in the PMA Ethiopia survey. Base map derived from the 2013 Ethiopian Central Statistical Agency (CSA) shapefile; survey zones merged using ArcGIS. Red circles mark sampled enumeration areas. (For interpretation of the references to colour in this figure legend, the reader is referred to the web version of this article.)

The study was conducted in Ethiopia, a Sub-Saharan African country with 74 administrative zones. The secondary data were obtained from the Performance Monitoring for Action (PMA) project, a global initiative led by the Johns Hopkins Bloomberg School of Public Health in partnership with Addis Ababa University, Ministry of Health, and the Ethiopian Central Statistical Agency (ECSA). A cluster sampling design (two-stage), stratified by urban–rural residence and major regions, and was implemented to ensure national representativeness. Enumeration areas (EAs) were first identified from the ECSA master frame. Within each EA, households were listed and then randomly selected (typically 35 per EA). Informed consent was obtained from all eligible women involved in the survey. The surveys were conducted through face-to-face interviews by trained female resident enumerators- local women over 21 with at least a high school diploma, who received two to three weeks of supervised training on protocols, ethics, and survey tools from regional coordinators and PMA Ethiopia staff. The household, female, and service delivery point (SDP) standardized questionnaires were used. Open Data Kit (ODK) and Android smartphones were used to program and administer these questionnaires. These instruments were adapted from the PMA2020 model and the Ethiopian Demographic and Health Survey (EDHS). The questionnaires addressed topics such as fertility intentions, contraceptive knowledge and use, reasons for non-use, unmet need for family planning, and additional reproductive health indicators. The complete questionnaires and technical documentation are available in the PMA Ethiopia Round 5 and Round 6 indicators ((PMA) 2017, (PMA) 2018). To generate the study map shown in Fig. 1, survey zones from PMA Ethiopia rounds 5 and 6 were combined with the 2013 Ethiopian administrative shapefile, obtained from the Ethiopian Central Statistical Agency, using ArcGis.

#### Variables

##### Outcome variable

The outcome variable for this study is women's FP status measured at the individual level. We generated a multinomial outcome variable for women's FP status by assigning 0 to women with neither unmet need nor contraceptive use, 1 to women who had an unmet need for family planning (i.e. want to delay or avoid pregnancy but were not using any contraceptive method), and 2 to women who were using any contraceptive method.

##### Predictor variables

Continuous and categorical variables were used to predict women's family planning status, including socio-demographic, maternal, service delivery factors, and spatial factors. The continuous variables were family size, age at first sex, service delivery days for FP, number of modern FP methods women heard, and geographic coordinates (longitude and latitude). The categorical variables included visit to a health center, desire for children, wealth status, visited by a health worker, education status, service delivery facility type, religion, delivery days open, sex avoidance, parity and media exposure (Table 1).

**Table 1:** Characteristics of study participants by continuous and categorical predictors of women's family planning status in Ethiopia, grouped into service delivery, maternal, socioeconomic/household, and religion categories.

Category	Variable	Categories / Unit	Description
Female household characteristics	Family size	count	Number of household members
	Age	years	Age of respondents
	Age at first sex	Years	Age when first sexual intercourse occurred
	Service delivery days for FP	Days per week	Number of days FP services available
	Number of modern FP methods heard	Count	Number of modern FP methods known
Service delivery characteristics	Visit to a health center	Yes / No	Whether respondent visited a health center
	Visited by a health worker	Yes / No	Contact with FP health worker
	Service delivery facility type	Public / Private / Hospital	Type of facility providing FP services
	Delivery days open	≤5 days / ≥6 days	Weekly availability of FP services
Maternal characteristics	Parity	0 / 1–2 / ≥3	Number of living children
	Desire for children	Yes / may be(NO)	Fertility preference
	Sex avoidance	Capable / Somewhat capable / Not capable	Ability to refuse sexual intercourse
Socio demographic characteristics	Education status	None / Primary / Secondary / Higher	Highest level of education attained
	Wealth status	High / Medium / Low	Household socioeconomic status (asset index)
	Religion	Orthodox/ Protestant and others/ Muslim	

### Statistical analysis

#### Model specification

The model employed in this study is a Geo-additive generalized linear mixed model, which extends the traditional GLM framework by incorporating spatially structured random effects and nonlinear smooth terms [13]. For a given covariate ω and unknown parameters ϕ, the distribution of the response variable y belongs to an exponential family with mean $\mu =E\left(y|u,\phi \right)$ linked to a linear predictor η by

$$\mu =h\left(\eta \right)=\omega_{i}^{\prime }\phi .$$

Here, h is a known response function, and ϕ are unknown regression parameters. Bayes X is, however, able to estimate much more flexible models with structured additive predictors;

$$\eta_{i}=f_{1}\left(x_{i1}\right)+\ldots +f_{p}\left(x_{ip}\right)+\omega_{i}^{'}\phi ,$$

where i is a generic observation index, $x_{ip}$ denotes generic covariates of different types and dimensions, and $f_{p}$ are smooth functions of the covariates. The functions $f_{p}$ comprise nonlinear effects of continuous covariates and spatially correlated effects.

When geographic information is available for the observations in the dataset, a suitable predictor should incorporate a spatially correlated effect, and can be expressed as follows:

$$\eta_{i}=f_{1}\left(x_{i1}\right)+\ldots +f_{p}\left(x_{ip}\right)+f_{\mathrm{spat}}\left(s_{i}\right)+\omega_{i}^{'}\phi$$

Where $f_{\text{spat}}$ is an additional spatially correlated effect of the location $s_{i}$ that an observation pertains to. Models with a predictor that contains a spatial effect are also called geo-additive models [14].

Because of spatial correlation and unobserved spatial heterogeneity in spatial data, the spatial effect is best decomposed into a structured, spatially correlated component and an unstructured, spatially uncorrelated component.

Since the spatial locations are clustered in connected geographical regions, a Markov Random Field prior is selected for the structured spatial effects. This spatial smoothness prior is defined by

$$\left\{f_{\mathrm{str}}\left(s\right)|f_{\mathrm{str}}\left(s^{\prime }\right);s\neq s^{\prime },\tau^{2}\right\}∼N\left(\sum_{s\in \delta_{s}}\;\;\frac{f_{\mathrm{str}}\left(s^{\prime }\right)}{N_{s}},\frac{\tau^{2}}{N_{s}}\right),$$

where $N_{s}$ is the number of adjacent sites, $s\in \delta_{s}$ indicates that site $s^{\prime }$ is a neighbour of site s, that is, they share a common boundary [15], [16].

The unstructured spatial effects are assumed to be i.i.d. random effects $f_{\text{unstr}}\left(s\right)∼$ $N\left(0,\tau^{2}\right)$.

A tensor product of two-dimensional p-splines was used to model the spatial effect, which is defined as:

$$f_{\text{spat}}\left(x_{1},x_{2}\right)=\sum_{i}^{k}\;\sum_{j}^{k}\;B_{\text{spat},\mathrm{ij}}B_{1i}\left(x_{1}\right)B_{2j}\left(x_{2}\right)$$

Where (x1, x2) denotes the geographical coordinates of the data. The function $f_{\text{spat}}\left(x_{1},x_{2}\right)$ represents the effects of correlated location factors that are not measured or observed. The prior assigned to $B_{\text{spat},i\;j}=\left(B_{\mathrm{spat},11},B_{\mathrm{spat},12},\ldots ,B_{\mathrm{spat},\mathrm{kk}}\right)$ follows spatial smoothness assumptions typical in spatial statistics. The study employed the most commonly used prior specification based on the four nearest neighbours.

#### Model selection and fit

The study compared three models to estimate fixed, nonlinear, and spatial effects of women's family planning choices. Model 1, a standard multinomial logistic regression model, included only the fixed effects of all covariates. Model 2, an additive model, incorporated fixed effects for categorical variables and nonlinear effects for continuous covariates using smoothing functions. Model 3, a Geo-additive model, further included a spatial random effect at the zonal level to capture spatial dependence. By comparing these models, the study evaluated how accounting for non-linearity and spatial structure improves model fit and interpretation, especially in revealing the spatial patterns underlying women's family planning behaviors. The estimated models are:

Model 1: $\eta_{i}$ = $\omega_{i}^{\prime }\phi$ (Multinomial logistic regression model).

Model 2: $\eta_{i}=f_{1}\left(x_{i1}\right)$ + $f_{2}\left(x_{i2}\right)$ + $\cdots +f_{p}\left(x_{\mathrm{ip}}\right)+\omega_{i}^{\prime }\phi \;$ (Additive model).

Model 3: $\eta_{i}=f_{1}\left(x_{i1}\right)$ + $f_{2}\left(x_{i2}\right)$ + $\cdots +f_{p}\left(x_{\mathrm{ip}}\right)$ + $f_{\mathrm{spa}t}\left(s_{i}\right)$ + $\omega_{i}^{\prime }\phi$ (Geo-additive model).

Where $\eta_{i}$ is the linear predictor of women's family planning status on different covariates, ϕ is unknown parameters (fixed effect parameter) corresponding to the categorical predictor, $f_{1}$…$f_{p}$ are nonlinear smooth effects of the continuous covariates, and $f_{\mathrm{spa}t}$ is the spatial correlation effect of the location.

After selection, the model fit was assessed using the Akaike Information Criterion (AIC), generalized cross-validation (GCV), and Log-likelihood (LogLik). The smaller the values of AIC and GCV and the higher the logLik value, the better the model fit.

#### Parameter estimation

The estimation procedure followed an empirical Bayes inference framework, where all variance or smoothing parameters were treated as unknown constants and estimated using the (approximate) restricted maximum likelihood method. No prior values were assigned to these parameters. The diffuse priors were assigned to fixed effects while obtaining unknown functions and covariate effects as posterior mode estimators by maximizing the posterior density given the estimated smoothing parameters.

### Spatial weight matrix

In this study, we used a queen contiguity-based spatial weight matrix. A spatial weight matrix, W, represents the degree of possible interaction between spatial areal units [17]. Specifically, a contiguity matrix encodes which zones share a common boundary or vertex, assuming that geographically proximate zones are more likely to exhibit similar family planning status due to shared socio-cultural, economic, or health service factors. By modeling spatial effects using a Markov Random Field approach, the analysis accounts for similarities between neighboring zones, reducing bias from geographic clustering and producing more reliable and interpretable estimates for family planning outcomes. The spatial weight matrix W with a binary variable is represented as follows:

$W_{\mathrm{ij}}$ =$\left\{\begin{array}{c} \;1\;\text{if area}\;i\;\text{and}\;j\;\mathrm{are}\;\text{neighbours};\;\\ 0\;\text{otherwise}.\;\; \end{array}\right)$

The binary spatial weights matrix W has a symmetric form. If W is row-standardized:

${W^{*}}_{\mathrm{ij}}$ =$\frac{W_{\mathrm{ij}}}{\sum_{j\in J}W_{\mathrm{ij}.}}$

where J is the set that includes all areal units that are contiguous with i. ${W^{*}}_{\mathrm{ij}}\;$ satisfies nonnegative ${W^{*}}_{\mathrm{ij}}$ ≥ 0 and $\sum_{j\in J}{W^{*}}_{\mathrm{ij}}$ =1 (for all *i* = 1, …, n). The binary weight is often used in spatial autocorrelation analysis with regular and irregular areal units.

### Statistical software

Analyses were performed in R using R2BayesX (v1.1–6), which interfaces with the BayesX software for structured additive regression modeling and spatial map objects were handled using shapefile functions from R2BayesX and the sp. package (v1.6–1).

## Results

### Summary of socio-demographic, maternal, and service delivery characteristics

The study had a complete response from all 7484 Ethiopian women between 15 and 49 years old. Participants indicated an average age of 29, a family size of 4.87, and 5.4 days for service delivery. The biggest group was Orthodox women, comprising 51.2%, with Muslims at 29.5%. Approximately 38.2% of people had no schooling, whereas 34.5% finished primary school. Most participants (58.6%) were wealthier, and more than half (58.3%) were childless. 73.3% desired more children, and approximately 51% reported no media consumption. Two-thirds (66%) had visited a health center in the reference period (Table 2).

**Table 2:** Summary of socio-demographic, maternal, and service delivery characteristics of women aged 15–49 (*N* = 7484, Ethiopia, 2017–2018).

Variable	Category	Frequency %	Variable	Category	Frequency %
Parity	Zero between 1 & 2 three or more	4365 (58.32) 1380(18.44) 1739 (23.24)	Sex avoid	Not at all capable Somewhat capable Capable	1567 (20.94) 4065 (54.32) 1852 (24.75)
Religion	Muslim orthodox protestant & others	2208 (29.50) 3829(51.16) 1447 (19.34)	Visited a health center	No Yes	2528 (33.78) 4956 (66.22)
Educational status	Never primary secondary& above	2862(38.24) 2578(34.45) 2044 (27.31)	Media exposure	No Yes	3817 (51.00) 3667 (49.00)
Wealth status	High Medium Low	4384 (58.58) 969 (12.95) 2131 (28.47)	Service delivery facility obtained	Other public hospital other private	5217 (69.71) 1747(23.34) 520(6.95)
Desire for children	Yes Maybe/no	5489 (73.34) 1995 (26.66)	Service delivery days opened	at most 5 days at least 6 days	3620 (48.37) 3864 (51.63)

Summary of continuous variables					
Variable	Age	Age at first sex	Family size	SDFP days	The number of modern FP methods women have heard about
Mean	29.36	17.34	4.872	5.4	7

### Model comparison

The geo-additive model outperformed both the additive and linear models, with the lowest AIC, GCV, and log-likelihood values, which shows the significance of spatial effects on women's FP status (Table 3).

**Table 3:** Comparison of linear, additive, and geo-additive models (*N* = 7484, Ethiopia, 2017–2018).

	AIC	GCV	LOGLIK
Linear Model	13,895	1.82171	−6901.5
Additive Model	13,823.4	1.80143	−6851.2
Geo-Additive model	13,408	1.68371	−6556.45

After model selection, we compared spline types and basis degrees. The P-spline with a first-order penalty (degree 2) performed best (Table 4).

**Table 4:** Comparison of Spline Basis Functions in the Geo-Additive Model (*N* = 7484, Ethiopia, 2017–2018).

	AIC	GCV	logLik
Second-order random walk	13,410	1.69187	−6568.45
P-spline with first-order penalty (degree 2)	13,408	1.68371	−6556.45
P-spline with first-order penalty (degree 3)	13,408	1.68493	−6558.15
P-spline with second-order penalty(degree 2)	13,411.7	1.69183	−6568.95
P-spline with second-order penalty (degree 3)	13,413.4	1.6926	−6570.55

### Estimates of the geo-additive model

Several covariates were associated with contraceptive use. Women's contraceptive use was negatively associated with desire for children (β = −1.77, 95% CI: -1.77 to -1.59).). In contrast, primary and higher levels of education (β = 0.31, 95% CI: 0.16 to 0.47; β = 0.27, 95% CI: 0.06 to 0.49) were positively associated with contraceptive use. Affiliation with Orthodox (β = 0.50, 95% CI: 0.33 to 0.68), Protestant and other religions (β = 0.38, 95% CI: 0.15 to 0.6), high wealth status (β = 0.46, 95% CI: 0.28 to 0.64), and visiting a health center (β = 0.17, 95% CI: 0.04 to 0.3) were also positively associated with contraceptive use. Women with three or more children were less likely to utilize contraception (β = −0.23, 95% CI: −0.43 to −0.04). Women's unmet needs were negatively associated with higher parity (≥3) (β = −0.26, 95% CI: −0.47 to −0.04), private health facility delivery (β = −0.59, 95% CI: −0.95 to −0.23), and desire for children (β = −1.88, 95% CI: −2.08 to −1.68). In contrast, the perceived ability to avoid sex was positively associated (β = 0.29, 95% CI: 0.07 to 0.51).

The structured spatial variance for contraceptive use was 0.92 and unstructured 0.07 (total = 0.99), so the structured component explained 93% of the variability. For unmet needs, the structured effect explained 68%, and the unstructured effect accounted 32% (Table 5).

**Table 5:** Estimated Parametric and Spatial Effects of the Geo-additive Model (*N* = 7484, Ethiopia, 2017–2018).

Variable	Category	Nonuse (n)	Unmet Needs (n)	Contraceptive (n)	Total (n)	Parametric Coefficients	Unadjusted β (95% CI)	Adjusted β (95% CI)	*p*-value
desire for children	May be/no	207	656	1132			Ref		
	Yes	2048	907	2534		Contraceptive	-1.70 (-1.71, -1.26)	-1.77 (-1.96, -1.59)	< 0.0001
						Unmet needs	-1.97 (-2.22, -1.72)	-1.88 (-2.08, -1.68)	<0.0001
education status	No education	1034	757	1071	2862	No education	Ref		
	Primary	701	531	1346	2578	Contraceptive-	0.61 (0.46, 0.77)	0.31 (0.16, 0.47)	0.0001
						Unmet needs	0.03 (-0.15, 0.21)	0.19 (0.01, 0.37)	0.04
	Secondary+	520	275	1249	2044	Contraceptive	0.84 (0.67, 1.01)	0.27 (0.06, 0.49)	0.01
						Unmet needs	-0.33 (-0.54, -0.11	0.09 (-0.16, 0.34)	0.5
facility delivered	Hospital	460	378	909	1747	Hospital	Ref		
	other private	163	74	283	520	Contraceptive	-0.13 (-0.41, 0.15)	-0.22 (-0.52, 0.08)	0.2
						Unmet needs	-0.59 (-0.98, -0.21)	-0.59 (-0.95, -0.23)	0.001
	other public	1632	1111	2474	5217	Contraceptive	-0.27 (-0.43, -0.1)	-0.08 (-0.24, 0.08)	0.3
						Unmet needs	-0.19 (-0.38, 0.01)	-0.11 (-0.3, 0.08	0.3
media exposure	no_exposure	1266	892	1659	3817		Ref		
	Atleast 1 exposure	989	671	2007	3667	Contraceptive	0.44 (0.29, 0.59)	-0.01 (-0.15, 0.13)	0.9
						Unmet needs	-0.04 (-0.22, 0.15)	0.08 (-0.08, 0.24)	0.3
parity	Zero	1326	864	2175	4365	Ref			
	between1and2	380	213	787	1380	Contraceptive	0.23 (0.06, 0.41)	0.14 (-0.04, 0.33)	0.1
						Unmet needs	-0.15 (-0.39, 0.08)	0.04 (-0.19, 0.28)	0.7
	threeormore	549	486	704	1739	Contraceptive	-0.25 (-0.41, -0.01)	-0.23 (-0.43, -0.04)	0.02
						Unmet needs	0.31 (0.12, 0.49)	-0.26 (-0.47, -0.04)	0.02
Religion	Muslim	1034	757	1071	2862	Ref			
	Orthodox	701	531	1346	2578	Contraceptive	1 (0.85, 1.16)	0.5 (0.33, 0.68)	< 0.0001
						Unmet needs	-0.07 (-0.25, 0.11)	0.03 (-0.18, 0.24)	0.8
	Protestant & others	520	275	1249	2044	Contraceptive	1.04 (0.84, 1.24)	0.38 (0.15, 0.6)	0.001
						Unmet needs	0.24 (0.01, 0.47)	0.13 (-0.13, 0.39)	0.3
sex avoid	not_capable	595	337	635	1567	Ref			
	Capable	486	391	975	1852	Contraceptive	0.63 (0.44, 0.83)	0.13 (-0.06, 0.32)	0.2
						Unmet needs	0.35 (0.12, 0.59)	0.29 (0.07, 0.51)	0.01
	some capable	1174	835	2056	4065	Contraceptive	0.5 (0.33, 0.66)	0.04 (-0.15, 0.23)	0.7
						Unmet needs	0.23 (0.03, 0.43)	-0.03 (-0.25, 0.19)	0.8
visited by health worker	No	1833	1277	2901	6011	Ref			
	Yes	422	286	765	1473	Contraceptive	0.14 (-0.05, 0.32)	0.02 (-0.14, 0.17)	0.8
						Unmet needs	−0.03 (-0.26, 0.21)	-0.10 (-0.29, -0.08)	0.3
visited a health center	No	826	628	1074	2528	Ref			
	Yes	1429	935	2592	4956	Contraceptive	0.33 (0.18–0.49)	0.17(0.04–0.3)	0.01
						Unmet needs	-0.34 (-0.15, 0.07)	-0.10 (-0.26, 0.04)	0.2
wealth status	Low	798	573	760	2131	Ref			
	High	1139	706	2539	4384	Contraceptive	0.85 (0.7, 1)	0.46 (0.28, 0.64)	<0.0001
						Unmet needs	-0.18 (-0.33, 0.03)	0.1 (-0.11, 0.29)	0.4
	Medium	318	284	367	969	Contraceptive	0.19 (-0.03, 0.42)	0.07 (-0.14, 0.27)	0.5
						Unmet needs	0.22 (-0.02, 0.46)	0.19 (-0.02, 0.41)	0.08
sdfp days	atmost5days	1143	766	1711	3620	Ref			
	atleast6days	1112	797	1955	3864	Contraceptive	0.16 (0.01, 0.31)	0.12 (-0.04, 0.29)	0.1
						Unmet needs	0.07 (-0.12, 0.25)	0.08 (-0.1, 0.25)	0.4
**Smoothing Effects**							**Variance**		
Contraceptive-structured spatial effect							0.92		
Unmet needs-structured spatial effect							0.21		
**Random Effects Variances**									
Contraceptive-unstructured effects							0.07		
Unmet needs-unstructured effects							0.0997		

**Fig. 2:**
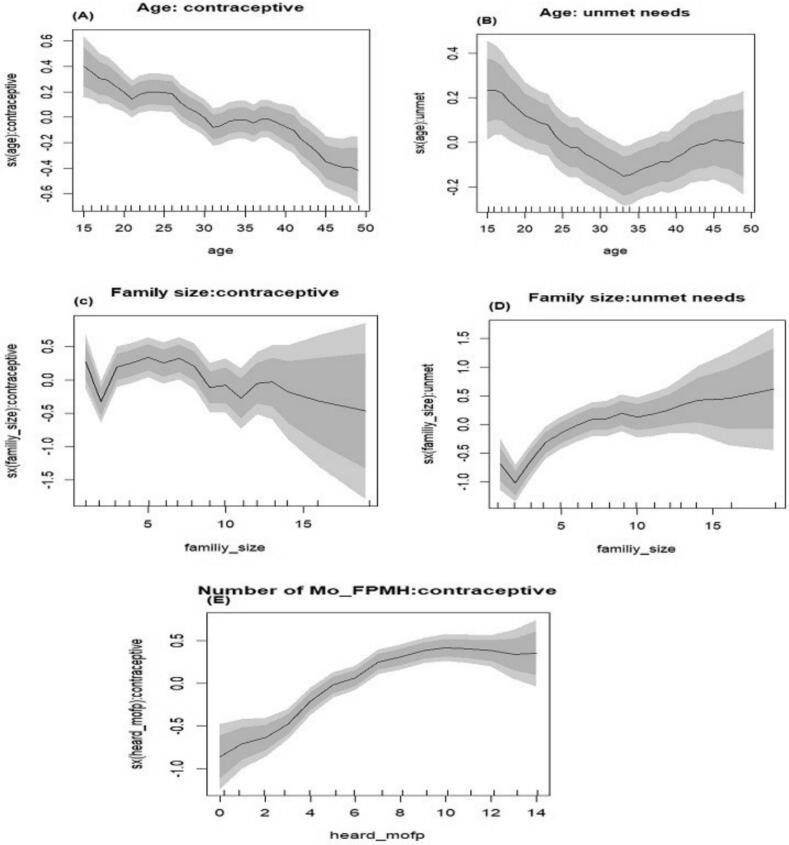
Estimated smooth effects of continuous covariates for contraceptive use and unmet needs categories.

The relationships between age, family size, and number of modern FP methods women heard about with contraceptive use and unmet needs were nonlinear (Fig. 2). Contraceptive use declined with age, highest among women aged 15–22 (Fig. 2 (A)). Clinically, this suggested that younger women were more likely to adopt contraception. In contrast, older women required additional counseling to maintain contraceptive use as access, health concerns, or method preference changes arise. Unmet need showed a V-shaped pattern: it decreased until about age 35, then rose, possibly because of reduced access, fewer method choices, or age-related health concerns (Fig. 2B).

**Fig. 3:**
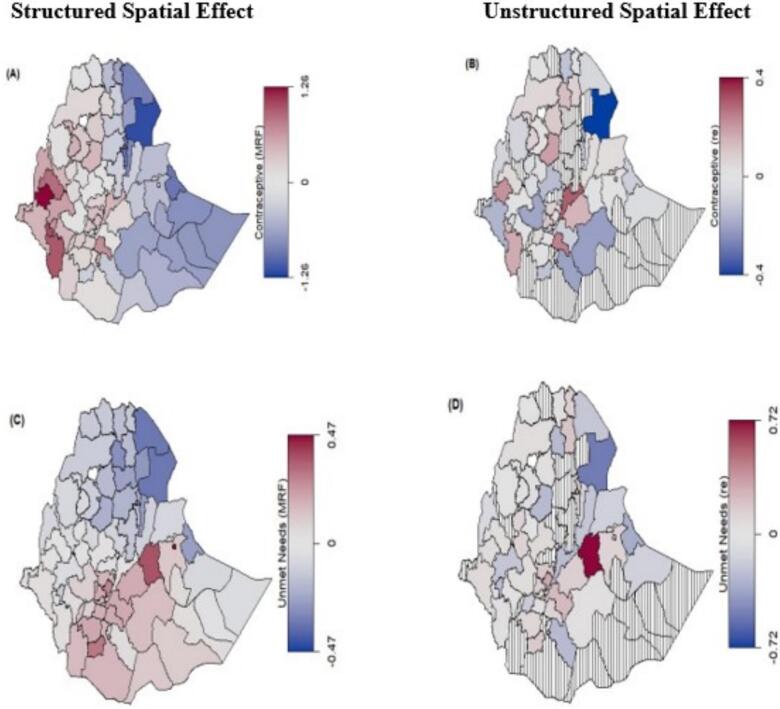
(A) shows marked geographic disparities in contraceptive use: lower prevalence in the northeast and southeast (darker blue), likely because of cultural and religious factors, and higher use in western zones (darker red), possibly linked to better service access and education. Fig. 3 (C) highlights central and southern areas with high unmet needs (deep red), contrasting with regions of lower unmet need (darker blue). The unstructured effects (Figs. 3 (B&D) reveal additional localized variations. (For interpretation of the references to colour in this figure legend, the reader is referred to the web version of this article.) Fig. 3:From left to right, averages estimates of the structured spatial effects (first column), unstructured Spatial effects (second column) for women's family planning choices.

Family size also influenced both outcomes (Fig. 2 (C & D)). Contraceptive use dipped among women with 2–3 children, then increased with larger families, suggesting greater demand for birth limitation. Conversely, unmet need rose steadily with family size, reflecting persistent access barriers. Finally, the number of modern FP methods known associated positively with contraceptive use, peaking at around 10 methods before levelling off, indicating diminishing returns from additional awareness. Clinicians should counsel women on a core set of methods most suited to their needs rather than overwhelming them with excessive options.

### Discussion

A geo-additive model was employed to study the complex relationships between individual, socioeconomic, and spatial factors influencing women's family planning status in Ethiopia. The results showed that geography affects these choices, underscoring the importance of location-specific interventions. Education, wealth, parity, health center visits, and childbearing preferences were associated with FP choices. Nonlinear effects of variables like age, family size, and exposure to FP methods further highlight the nuanced nature of FP decision-making. Structured spatial effect plays a significant role in Ethiopian women's FP choices, with a range of variation of 93% for contraceptive use and 68% for unmet needs.

Although, we used the PMA surveys conducted in 2017–2018, they provide a robust baseline for understanding historical patterns and spatial disparities in family planning. These nationally representative datasets, enriched with geo-referenced information, remain highly relevant for programmatic planning. Since 2018, Ethiopia has experienced demographic shifts, FP2030 policy reforms, expansion of health posts, and sociopolitical disturbances in certain regions. These changes may have influenced contraceptive dynamics, but the zonal variations identified in our study continue to serve as an important reference point for evaluating progress and guiding future interventions.

Women with a primary or above educational level have a positive effect on women contraceptive status. This may be because women's education leads to greater family planning knowledge, autonomy, and access to healthcare services. This finding is in line with studies conducted in Turkey, Ethiopia, and sub-Saharan Africa [18], [19].

Age showed linear relationship with women's contraceptive status, and it has a decreasing trend. This suggests that younger women are more likely to use contraception compared to older women, which may be due to greater exposure to FP messages or different fertility preferences. This result is in line with a study conducted in Rwanda [20], which also reported higher contraceptive use among younger women. This study revealed parity had an association with women contraceptive use. Women with more children were more likely to adopt contraception. This finding also aligns with the study conducted in Ethiopia and Indonesia [18], [21], [22], where higher parity was similarly associated with increased contraceptive uptake.

The findings from this study indicate that women's desire for more children negatively affects contraceptive use. In other words, women who desire to have additional children are less likely to use contraception. This result is consistent with studies conducted in Bangladesh and Pakistan [23], [24], which similarly found that higher fertility desires reduce contraceptive uptake.

This study also revealed that desire for children had an association with the women's unmet needs for FP status. Women who desire more children are less likely to use contraception, which increases their unmet needs for family planning. Because they are not using contraception, they are more likely to experience unintended or closely spaced pregnancies. This finding is in line with studies conducted in Ethiopia [25], [26]

The number of modern FP methods women heard had an association with women's contraceptive use. This finding aligns with studies conducted in Ethiopia and Ghana [12], [27]. Conversely, research in Jimma Zone, Ethiopia, indicates a substantial gap between knowledge and actual contraceptive practice, despite widespread awareness of FP. Barriers such as fear of side effects, health concerns, and socio-cultural influences hinder usage. In Nigeria, low contraceptive usage rates, despite high awareness, suggest that factors beyond knowledge, such as access to services and cultural acceptance, are critical to understanding contraceptive uptake [28].

According to our research, women who reported being able to avoid sex had a positive association with unmet need for family planning (β= 0.29, 95%CI 0.07 to 0.51)). This finding underlines the importance of women's control over their sexuality in determining their reproductive health. When women can negotiate sex or say no, they tend to have more control over their reproductive choices, which means fewer women have unmet needs. This finding is in line with studies from Ethiopia [29], and Ghana [30], found a positive link between women's autonomy, including the right to decline sex, and the use of reproductive health services.

According to study, religion has a substantial impact on contraceptive use. Orthodox Christians and Protestants/other groups reported higher rates of contraception use compared to Muslim women. This tendency is consistent with previous findings in Ethiopia, where contraceptive usage is lower among Muslim groups, typically due to cultural and religious norms encouraging bigger families and poorer acceptance of modern contraception [31], [32].

Women's desire for children was strongly associated with contraceptive use and unmet need. Women with three or more children were less likely to use contraception and had higher rates of unmet needs. This is in line with a systematic review conducted in Africa [33], which reported that fertility demand and parity are major factors influencing contraceptive uptake. This underscores the importance of parity and fertility preferences in shaping family planning outcomes. Overall, our findings emphasize that fertility intentions, family size, and socio-cultural influences are central to understanding reproductive health behaviors in Africa.

Our findings showed that media exposure was not associated with contraceptive use. This aligns with a recent study from Nigeria, which reported that mobile technology and social media exposure did not independently reduce abortion experience [34]

The prevalence of women's contraceptive use choice in eastern Ethiopia (Ethiopian Somali and Afar) is very low. This finding is similar to the study conducted in Ethiopia [12], [35]. The structural geographic effect reveals large zonal differences in Ethiopian women's FP status. Contraceptive use was lower in the eastern, and southeastern regions and higher in northern, central, and western Ethiopia. This result is similar to studies conducted in Ethiopia [12], [26], [36], [37], [38]. The low prevalence of contraceptive use may be due to cultural and religious beliefs. This is consistent with studies conducted in Ethiopia and the Democratic Republic of Congo, which revealed provincial/zonal variations in contraceptive use [39], [40].

The geo-additive model provides a more thorough comprehension than conventional logistic regression, which is constrained by its assumption of linear relationship and its inability to incorporate non-linear variables and spatial effects. Though additive models are flexible in nonlinear effects, they fail to account for spatial variation. The geo-additive model accounts for both nonlinear covariates and spatial effects. For Ethiopia, it is very important to use these models to address regional and zonal disparities that affect family planning. Studies have shown that geo-additive models enhance generalized linear models by incorporating spatial correlation, which increases precision and uncovers hidden geographic patterns [41], [42]

The strength of this study was in its use of advanced statistical methods to reveal zonal spatial variation in women's FP status and identify linear and non-linear covariates that play an essential role.

This study had certain limitations. Since the data covered only two years, we could not analyze the trend in family planning status over time. The short duration may have hindered our ability to detect temporal or long-term changes in contraceptive use. Even though the PMA survey was nationally representative, the analysis was limited to 53 out of Ethiopia's 74 administrative zones. This incomplete coverage may limit the spatial clustering pattern's ability to fully capture geographic heterogeneity across all zones. It did not include certain maternal, socio-cultural, and other environmental factors that were likely to influence family planning decisions. In our study, we treated contraceptive use as a single category, without accounting for specific methods, which may have contributed to some significant differences.

In the future, an extended study period and inclusion of all zones should be undertaken to assess spatial trends and dynamics and to capture the spatial heterogeneity of family planning across Ethiopian zones. Furthermore, to increase explanatory power and reduce bias, researchers should incorporate additional factors and differentiate contraceptive methods at the zonal level to enable more effective intervention tailoring.

## List of abbreviations

**Table:** 

AIC	Akaki Information Criterion
FP	Family Planning
GCV	Generalized Cross Validation
PMA	Performance Monitoring for Action
LogLik	Log likelihood
STAR	Structural Additive Regression Model

## CRediT authorship contribution statement

**Mandefro Abere Tegegne:** Writing – review & editing, Writing – original draft, Software, Methodology, Formal analysis, Data curation, Conceptualization. **Essey Kebede Muluneh:** Writing – review & editing, Writing – original draft, Validation, Supervision, Methodology, Formal analysis. **Aweke Abebaw Mitku:** Writing – review & editing, Writing – original draft, Validation, Supervision, Methodology, Formal analysis, Conceptualization. **Girma Taye Aweke:** Writing – review & editing, Writing – original draft, Validation, Supervision.

## Ethics statement

This study used publicly available secondary data obtained through an online request from the Performance Monitoring and Accountability 2020 (PMA2020) project website. The original data collection adhered to local legislation and the ethical standards set by the Ethiopian Central Statistical Agency. The primary data collectors obtained ethical approval and informed consent, with oversight from relevant national and institutional review boards. Additional ethical approval was not required for this secondary analysis, as the data were fully anonymized and accessible upon free registration. We acknowledge the PMA2020 project, the institutions involved, and the study participants for making the data publicly available. The approval email granting permission to access and use the data is attached as supplementary material.

## Declaration of generative AI and AI-assisted technologies in the writing process

During the preparation of this work, the author(s) used Grammarly to improve readability and language. After using this tool/service, the author(s) reviewed and edited the content as needed and took (s) full responsibility for the publication's content.

## Funding

This research received no specific grant from funding agencies in the public, commercial, or not-for-profit sectors.

## Declaration of competing interest

The authors declare that they have no known competing financial interests or personal relationships that could have appeared to influence the work reported in this paper.

The authors declare the following financial interests/personal relationships which may be considered as potential competing interests

## Data Availability

The datasets used in this study are publicly available on the PMA2020 project website https://www.pmadata.org/data/available-datasets. Specifically, the study utilized the PMA2020 Ethiopia Round 5 (2017) Household and Female Survey PMA2017/ET-R5-HQFQ, the Round 5 Service Delivery Point Survey PMA2017/ET-R5-SQ, the Round 6 (2018) Household and Female Survey PMA2018/ET-R6-HQFQ, and the Round 6 Service Delivery Point Survey PMA2018/ET-R6-SQ. These datasets can be accessed freely at https://doi.org/10.34976/0vv8-bc40 https://doi.org/10.34976/30MN-C910 https://doi.org/10.34976/EKX0-WF31 https://doi.org/10.34976/EK1B-WE38
